# Metastatic Lung Adenocarcinoma Mimicking Miliary Tuberculosis: A Case Report

**DOI:** 10.7759/cureus.88494

**Published:** 2025-07-22

**Authors:** Hiba J Merhi, Mohammad W Chamseddine, Abbas I Mhaidly, Zahraa K Al Najjar, Ali R Raad

**Affiliations:** 1 Pulmonary and Critical Care Medicine, Faculty of Medicine, Lebanese University, Beirut, LBN; 2 Internal Medicine, Faculty of Medicine, Lebanese University, Beirut, LBN; 3 General Medicine, Faculty of Medical Sciences, Lebanese University, Beirut, LBN; 4 Pathology, Al Rassoul Al-Aazam Hospital, Beirut, LBN; 5 Pulmonary and Critical Care Medicine, Al Rassoul Al-Aazam Hospital, Beirut, LBN

**Keywords:** adenocarcinoma, immunohistochemistry (ihc), lung adenocarcinoma, lung cancer, metastatic adenocarcinoma, miliary tuberculosis, mimics tuberculosis, pulmonary nodules, tissue biopsy

## Abstract

Lung adenocarcinoma (LA) can present with a wide range of morphological patterns and may mimic disseminated infectious diseases such as miliary tuberculosis (TB), posing significant diagnostic challenges and potentially delaying appropriate treatment. We report a case of metastatic pulmonary adenocarcinoma that was initially misdiagnosed as miliary TB. A 35-year-old nonsmoking male presented with a progressive dry cough and shortness of breath. Chest imaging revealed diffuse bilateral micronodules and a pericardial effusion, raising suspicion for miliary TB. Although acid-fast bacilli smears and a purified protein derivative test were negative, empiric anti-TB therapy was initiated based on radiographic findings. Despite treatment, the patient’s condition deteriorated. Further evaluation, including a cervical lymph node biopsy, unexpectedly revealed metastatic, moderately differentiated adenocarcinoma of pulmonary origin. A subsequent pericardial biopsy confirmed metastatic involvement. Anti-TB therapy was discontinued; however, the patient’s clinical status continued to decline. This case highlights the diagnostic challenge of metastatic LA mimicking miliary TB. In low TB-burden settings, it is essential to maintain a broad differential diagnosis and to consider alternative etiologies, such as metastatic malignancies, when confronted with miliary patterns on chest imaging, particularly in the absence of classic TB risk factors or poor response to treatment. Tissue biopsy from accessible sites and immunohistochemistry remain critical for establishing an accurate diagnosis and guiding appropriate management in such complex presentations. This case underscores the limitations of relying solely on imaging and reinforces the need for a thorough diagnostic workup when evaluating diffuse micronodular lung patterns.

## Introduction

Miliary tuberculosis (TB) is a severe form of TB resulting from the hematogenous dissemination of *Mycobacterium tuberculosis *throughout the body [1]. It often presents with nonspecific symptoms such as fever, weight loss, night sweats, and anorexia - clinical features that can mimic other diseases, particularly primary and secondary lung malignancies. Risk factors for miliary TB include immunosuppression due to cancer, HIV infection, malnutrition, diabetes, organ transplantation, and end-stage renal disease [2].

The clinical manifestations of TB are highly variable, making diagnosis particularly challenging [3]. A miliary pattern observed on chest imaging is not pathognomonic and may be seen in various other conditions, including occupational lung diseases, disseminated fungal infections, sarcoidosis, and metastatic disease from primary or secondary lung cancers. Therefore, CT findings alone are insufficient for a definitive diagnosis. Additional diagnostic workups, such as serological tests and histopathological examination of tissue specimens, are essential to establish the correct etiology [2,4,5].

Lung adenocarcinoma (LA) - the most common type of lung cancer, accounting for approximately 40% of all cases - occurs in both smokers and nonsmokers [5]. It is typically located in the peripheral lung regions and is often associated with chronic inflammation [6]. LA can present with diverse radiological appearances, including solid nodules, ground-glass opacities, or part-solid/part-ground-glass nodules [7]. Imaging may also reveal multifocal, peribronchovascular consolidations with air bronchograms - air-filled bronchi visible against surrounding opaque lung parenchyma [3]. Although rare, LA may also present with multiple discrete nodules resembling a miliary pattern, with only a few such cases reported in the literature [8].

Globally, both TB and LA remain major public health concerns. According to WHO, an estimated 10.8 million people developed TB in 2023 [9]. Meanwhile, lung cancer remains a leading cause of cancer-related morbidity and mortality. In 2022, there were an estimated 2,480,675 new cases of lung cancer worldwide, of which adenocarcinoma accounted for a substantial proportion - 717,211 new cases in men alone, representing 45.6% of the total male lung cancer burden [10].

The clinical and radiological overlap between pulmonary TB and LA often leads to diagnostic confusion [11]. Several case reports have documented instances where LA initially mimicked miliary TB, leading to misdiagnosis and delayed treatment. For example, Seifi et al. (2022) reported a case involving a 20-year-old man with metastatic LA initially presumed to have miliary TB based on clinical and radiological findings [2]. Similarly, Khan et al. (2020) described a 35-year-old Indian male who presented with a two-month history of dry cough and dyspnea; chest imaging revealed diffuse bilateral miliary nodules, initially thought to represent miliary pulmonary TB [12]. Such cases underscore the diagnostic challenges when metastatic LA presents with imaging features suggestive of miliary TB [2].

Pulmonary TB can obscure the presence of LA, resulting in delayed diagnosis and treatment. In the case presented here, a 35-year-old man was initially diagnosed with TB. However, due to poor treatment response and subsequent investigations, coexistent LA was identified.

## Case presentation

A 35-year-old male office worker, previously healthy with no history of smoking or known drug or food allergies, presented with a one-week history of progressive dry cough and shortness of breath. He denied fever, sputum production, vomiting, diarrhea, or other systemic symptoms. Physical examination was unremarkable, with no significant findings. He was afebrile, hemodynamically stable, and had an oxygen saturation of 94% on room air.

Initial evaluation at a rural hospital revealed leukocytosis with neutrophilic predominance and an elevated CRP. Other routine laboratory investigations, including renal and liver function tests, serum electrolytes, and troponin levels, were within normal limits (Table 1).

**Table 1:** Summary of routine blood investigations MCH, mean corpuscular hemoglobin; MCHC, mean corpuscular hemoglobin concentration; MCV, mean corpuscular volume; RBC, red blood cell count; RDW, red cell distribution width; SGOT, serum glutamic oxaloacetic transaminase; SGPT, serum glutamic pyruvic transaminase; WBC, white blood cell count

Laboratory parameter	Patient’s value	Reference range
MCV	82.7 fL	80-100 fL
RBC	6.06 million cells/µL	Males: 4.5-5.9 million cells/µL
Platelet count	335 × 10³/µL	150-450 × 10³/µL
MCH	27.7 pg	27-33 pg
MCHC	33.5 g/dL	32-36 g/dL
Basophils	0.20%	0-2%
Lymphocytes	13%	20-40%
Monocytes	6.80%	2-8%
RDW	34.90%	11.5-14.5%
Segmented neutrophils	79.50%	40-70%
Eosinophils	0.4 × 10⁹/L	0-0.5 × 10⁹/L
Sodium	139 mmol/L	135-145 mmol/L
Potassium	4.4 mmol/L	3.6-5.1 mmol/L
Chloride	105 mmol/L	96-106 mmol/L
SGOT	20 U/L	10-40 U/L
SGPT	22 U/L	7-56 U/L
Fasting glucose	89 mg/dL	70-99 mg/dL
Creatinine	0.89 mg/dL	0.7-1.3 mg/dL
Troponin	0.02 ng/mL	<0.04 ng/mL
CRP	60 mg/L	<10 mg/L
WBC	13.4 × 10⁹/L	4.5-11.0 × 10⁹/L
Hemoglobin	16.8 g/dL	Males: 13.5-17.5 g/dL
Hematocrit	50%	Males: 39-50%

**Figure 1:**
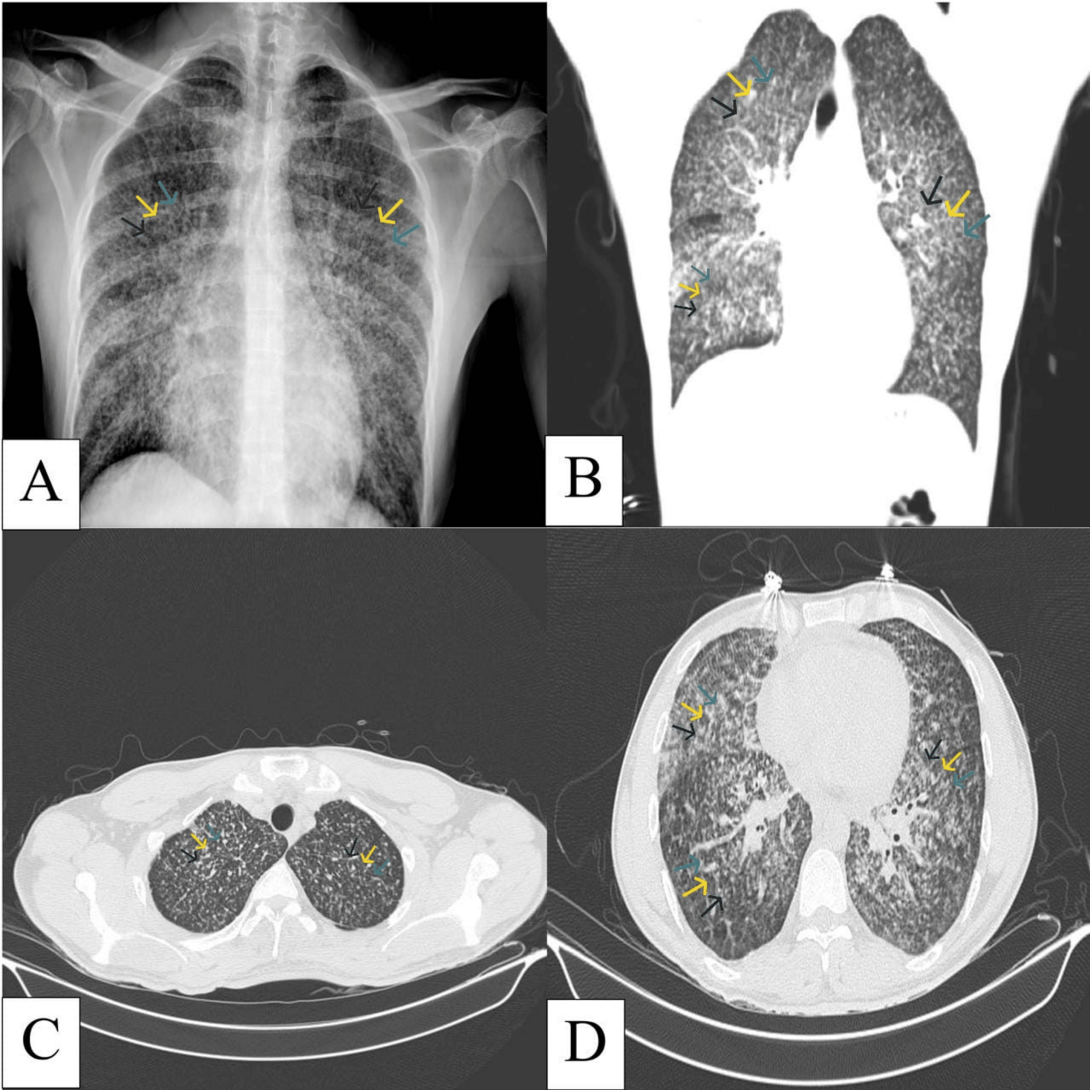
Chest radiography and CT images (A) Chest X-ray showing increased bronchovascular markings (arrows). (B) Coronal CT image of the chest showing diffuse pulmonary nodules measuring 1-2 mm (arrows). (C) Axial CT image showing diffuse pulmonary nodules in both upper lobes (arrows). (D) Axial CT image showing diffuse pulmonary nodules in the right middle and lower lobes, lingula, and left lower lobe (arrows).

A chest X-ray demonstrated increased bronchovascular markings (Figure 1A). A high-resolution CT scan of the chest revealed numerous, diffusely distributed 1-2 mm pulmonary nodules in both lungs (Figure 1B, 1C, 1D), raising suspicion for miliary TB.

Cardiac size was unremarkable; however, a moderate pericardial effusion was noted. Acid-fast bacilli smears and a purified protein derivative test were negative. Transthoracic echocardiography confirmed a moderate pericardial effusion measuring approximately 1.6 cm, with otherwise normal cardiac structure and function.

The patient was transferred to a tertiary care center after two days for further evaluation. Upon admission, he was hemodynamically stable, afebrile, and had an oxygen saturation of 90% on room air. Repeat laboratory investigations showed persistent leukocytosis and elevated CRP, while other parameters - including blood urea nitrogen, creatinine, liver enzymes, lactate dehydrogenase, erythrocyte sedimentation rate, and procalcitonin - remained within normal limits. Influenza and SARS-CoV-2 antigen tests were negative.

Extensive serological testing for autoimmune and infectious etiologies - including antinuclear antibodies, complement levels (C3 and C4), antineutrophil cytoplasmic antibodies, anti-double-stranded DNA antibodies, angiotensin-converting enzyme, adenosine deaminase, HIV, and hepatitis B and C serologies - were all negative. A repeat chest X-ray showed diffuse interstitial nodularity.

A subsequent chest CT confirmed a diffuse micronodular pattern with mediastinal lymphadenopathy and a moderate pericardial effusion. Based on these radiographic findings, empiric anti-TB therapy was initiated.

CT of the abdomen and pelvis revealed normal-appearing kidneys, tiny calcifications in the left adrenal gland, and no evidence of organomegaly, ascites, or intra-abdominal lymphadenopathy. Bronchoscopy showed normal bronchial mucosa without visible endobronchial lesions. Bronchoalveolar lavage fluid analysis was positive for *Streptococcus pneumoniae *and *Legionella pneumophila *on a multiplex respiratory pathogen panel. The patient was treated with ceftriaxone 2 g intravenously once daily for five days and azithromycin 500 mg orally once daily for three days, targeting these organisms.

Repeat echocardiography demonstrated a moderate-to-large pericardial effusion (~2.4 cm) with preserved ejection fraction. Pericardiocentesis was performed; however, cultures of the pericardial fluid were negative.

After two weeks of anti-TB treatment (isoniazid, rifampin, ethambutol, and pyrazinamide) and antibiotics, the patient’s condition worsened, with persistent dyspnea, decreased oral intake, and increased oxygen requirements. He was referred to our institution for consideration of a pericardial window. Physical examination revealed a thin, anicteric, nonpale male with a soft, nontender abdomen; clear lungs on auscultation; normal heart sounds without murmurs; no lower limb edema; and palpable cervical lymph nodes.

**Figure 2:**
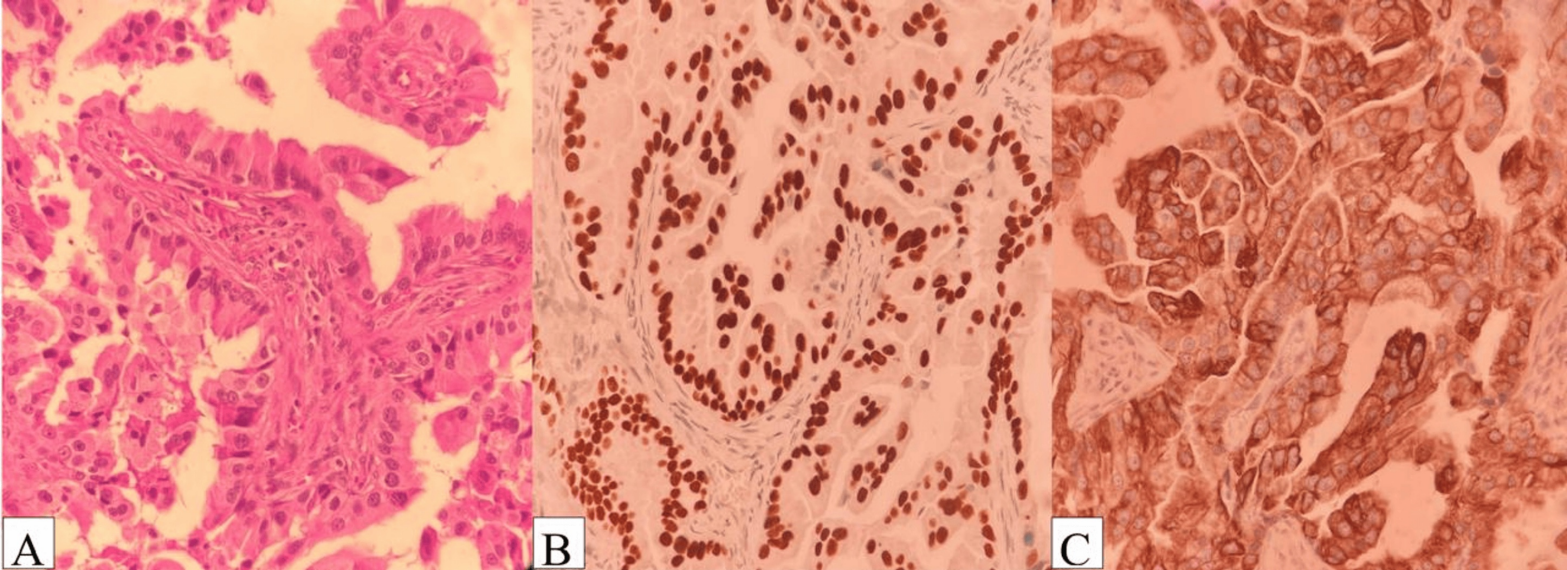
Immunohistochemistry imaging (A) H&E staining showing metastatic lymph node involvement. (B) Immunohistochemistry staining positive for cytokeratin 7, demonstrating membranous positivity in tumor cells. (C) Immunohistochemistry staining positive for thyroid transcription factor 1, showing nuclear positivity in tumor cells.

A cervical lymph node biopsy revealed metastatic, moderately differentiated adenocarcinoma. One week later, immunohistochemical analysis confirmed the diagnosis of pulmonary papillary adenocarcinoma, with tumor cells expressing CK7 and TTF1 and negative for CK20, CDX2, PAX8, and thyroglobulin (Figure 2A, 2B, 2C). A subsequent pericardial biopsy also demonstrated infiltration by moderately differentiated adenocarcinoma cells.

The patient continued to deteriorate clinically, requiring high-flow nasal cannula support due to worsening hypoxemia. Following the definitive diagnosis, anti-TB therapy was discontinued, and molecular testing was promptly ordered. This step was critical for several reasons: to identify specific genetic alterations within the tumor, guide targeted therapy, avoid unnecessary chemotherapy, improve prognosis, and determine the appropriateness of immunotherapy.

The patient was subsequently transferred to another hospital to initiate chemotherapy with pemetrexed and cisplatin, along with immunotherapy using pembrolizumab, while awaiting the results of molecular testing - including EGFR mutation, ALK rearrangement, ROS1, RET, BRAF, MET, KRAS, and NRAS - and immunohistochemistry studies such as PD-L1 expression, HER2, and microsatellite instability. Ten days later, the molecular and immunohistochemistry results were reported as negative, with 0% tumor cells staining positive for PD-L1. As a result, the patient was continued on the initially prescribed regimen.

## Discussion

The presented case highlights a significant diagnostic challenge: a rare scenario in which metastatic pulmonary adenocarcinoma mimicked the radiological features of miliary TB. The initial diagnostic confusion underscores the frequently overlooked overlap between miliary TB and other disseminated lung diseases, including metastatic malignancies. Specifically, the miliary pattern is a subcategory of the nodular pattern, characterized by numerous, diffusely distributed, well-defined micronodules of uniform size (1-2 mm), resembling millet seeds [13]. This finding is consistent with previously documented cases in the literature, including a case series [14] and several individual case reports [2,3,5,15].

However, this case goes beyond mere description. It emphasizes the limitations of relying solely on pattern recognition without thorough clinical correlation and the necessity of aggressively pursuing a definitive diagnosis, particularly in atypical presentations. While diffuse pulmonary micronodules on chest CT are a hallmark of miliary TB, this pattern is not pathognomonic and may also be seen in various other conditions, such as occupational lung diseases, fungal infections, and metastatic cancer [1].

This diagnostic ambiguity is particularly critical in regions like Lebanon, a country with a low TB burden. Although empirical anti-TB treatment is often initiated in patients presenting with nonspecific symptoms and miliary shadows, this case strongly advocates for maintaining a high index of suspicion for alternative etiologies - particularly malignancy - when the clinical presentation is atypical or when empiric therapy fails to produce improvement [10].

The presence of moderate pericardial effusion further complicated the diagnostic picture, as it is a common finding in both TB and advanced malignancy. This overlap contributed to the initial clinical misdirection. The initial diagnostic workup, including negative infectious and autoimmune serologies, played a pivotal role in refining the differential diagnosis. Although bronchoalveolar lavage revealed *S. pneumoniae* and *L. pneumophila*, the lack of clinical improvement despite targeted antibiotic therapy raised significant concern. The absence of therapeutic response strongly suggested that these organisms were either colonizers or secondary pathogens, not the primary cause of the diffuse pulmonary infiltrates. This prompted further investigation into a more insidious underlying etiology.

Persistent leukocytosis and elevated CRP, though nonspecific, pointed toward an ongoing inflammatory or neoplastic process that required further clarification. The turning point in this case was the cervical lymph node biopsy, which unexpectedly revealed metastatic adenocarcinoma. This result prompted a pericardial biopsy, which confirmed metastatic involvement. Immunohistochemical analysis provided molecular insights that strongly supported a primary pulmonary origin of the adenocarcinoma. This comprehensive diagnostic approach ultimately clarified the underlying etiology behind both the miliary-like radiographic pattern and the pericardial effusion, resolving what had initially been a highly ambiguous presentation.

In the era of precision medicine, immunohistochemistry plays a critical role in classifying tumors into specific subtypes and identifying biomarkers that enable rapid and accurate treatment decisions [16]. The CK7 and CK20 immunoprofile is useful in differentiating pulmonary, ovarian, and breast carcinomas (CK7+/CK20−) from colon (CK7−/CK20+), urothelial (CK7+/CK20+), and renal or prostatic carcinomas (CK7−/CK20−) [17]. When a CK7+/CK20− profile is established, additional markers such as TTF-1, GATA3, PAX8, and WT1 help narrow the differential diagnosis. For example, a profile of TTF-1+, Napsin A+, and GATA3− supports the diagnosis of LA - consistent with our patient, who was CK7+/CK20−/TTF-1+ [18].

LA is the most histologically diverse form of lung cancer, encompassing multiple subtypes with overlapping features [6]. In its early stages, it is often asymptomatic. As the disease progresses, symptoms such as cough, hemoptysis, weight loss, and pleural effusion typically emerge. Less common presentations include superior vena cava syndrome, pericardial effusion, Horner syndrome, and various paraneoplastic syndromes [6]. The atypical miliary pattern observed in our patient reflects the protean manifestations of LA and underscores how this common malignancy can mimic less prevalent diseases, creating substantial diagnostic challenges.

Although WHO’s classification of lung tumors continues to evolve, it still faces limitations in addressing the full spectrum of complex biological factors necessary for a precise diagnosis - an issue that may lead to misclassification and impact clinical management [19]. Intraoperative frozen section analysis remains an important tool but often results in indeterminate diagnoses, particularly in distinguishing between minimally invasive and invasive adenocarcinomas [20]. This diagnostic complexity at the pathological level highlights the importance of a multimodal approach - integrating clinical, radiological, and histopathological data - as demonstrated in this case. Accurate subtyping is critical for guiding treatment strategies and assessing prognosis, given that different subtypes vary in aggressiveness and treatment response. Despite significant advances in diagnostic tools, LA remains a formidable diagnostic challenge, necessitating ongoing refinement of classification systems and diagnostic strategies.

This case offers several important clinical lessons for practicing physicians. First, in patients presenting with a miliary pattern on chest imaging - particularly in the absence of strong epidemiological risk factors for TB or with a negative initial infectious workup - a broad differential diagnosis, including metastatic malignancies, must be thoroughly considered. Second, a lack of clinical or radiological improvement following empiric anti-TB therapy should prompt immediate re-evaluation of the diagnosis and a proactive search for alternative etiologies. Treatment failure in this context is a critical diagnostic clue that should not be overlooked. Third, tissue biopsy from accessible sites - such as lymph nodes or the pleura/pericardium when an effusion is present - plays an essential and often definitive role in establishing an accurate diagnosis in complex or ambiguous cases, thereby guiding timely and appropriate management. This case highlights the importance of diagnostic persistence and a systematic approach to atypical presentations to avoid misdiagnosis and improve patient outcomes.

## Conclusions

A miliary pattern on chest imaging presents a significant diagnostic challenge, as it may be associated with a wide range of conditions, from benign infections to serious malignancies such as metastatic LA, as demonstrated in this case. Misdiagnosis poses substantial risks, including delays in initiating appropriate treatment for potentially life-threatening diseases, ultimately compromising patient outcomes. As such, maintaining a broad differential diagnosis is crucial, particularly when confronted with atypical clinical presentations.

This case underscores the importance of maintaining a high index of suspicion and pursuing a definitive diagnosis through timely tissue biopsy when a miliary pattern is identified. A proactive, investigative approach is essential to ensure appropriate and timely management, especially in instances where metastatic LA mimics other conditions, thereby avoiding the serious consequences of delayed or incorrect diagnosis.
